# Relationship Between Myo-Inositol Supplementary and Gestational Diabetes Mellitus: A Meta-Analysis

**DOI:** 10.1097/MD.0000000000001604

**Published:** 2015-10-23

**Authors:** Xiangqin Zheng, Zhaozhen Liu, Yulong Zhang, Yuan Lin, Jianrong Song, Lianghui Zheng, Sheng Lin

**Affiliations:** From the Daoshan road 18, Gulou District, Department of Obstetrics and Gynecology, Fujian Provincial Maternal and Child Health Hospital, Fujian Medical University Teaching Hospital, Fuzhou 350000, Fujian, China.

## Abstract

To determine whether myo-inositol supplement will increase the action of endogenous insulin, which is mainly measured by markers of insulin resistance such as homeostasis model assessment of insulin resistance.

PubMed, Cochrane Library, Embase, and web of science were comprehensively searched using “gestational diabetes mellitus” and “myo-inositol” to identify relevant studies. Both subject headings and free texts were adopted. The methodological quality of the included studies were assessed and pooled analyzed by the methods recommended by the Cochrane collaboration.

A total of 5 trials containing 513 participants were included. There was a significant reduction in aspects of gestational diabetes incidence (risk ratio [RR], 0.29; 95% confidence interval (95% CI), 0.19–0.44), birth weight (mean difference [MD], −116.98; 95% CI, −208.87 to −25.09), fasting glucose oral glucose tolerance test (OGTT) (MD, −0.36; 95% CI, −0.51 to −0.21), 1-h glucose OGTT (MD, −0.63; 95% CI, −1.01 to −0.26), 2-h glucose OGTT (MD, −0.45; 95% CI, −0.75 to −0.16), and related complications (odds ratio [OR], 0.28; 95% CI 0.14–0.58).

On the basis of current evidence, myo-inositol supplementation reduces the development of gestational diabetes mellitus (GDM), although this conclusion requires further evaluation in large-scale, multicenter, blinded randomized controlled trials.

## INTRODUCTION

Gestational diabetes mellitus (GDM) is a complication of pregnancy, defined as carbohydrate intolerance at the onset of pregnancy or first recognized during pregnancy.^1^ It has long been regarded as incurring an increased risk of pregnancy-related maternal and perinatal morbidity and long-term adverse outcomes for women with GDM and their children.^2^ The prevalence of GDM is increasing,^3^ with almost 10% of pregnancies complicated by it, and its prevalence may double with the newly proposed criteria for the diagnosis of GDM.^4^ Therefore, safe, effective, acceptable, and simple interventions to prevent GDM are required, but until now, no systematic reviews have provided conclusive evidence of successful interventions to prevent GDM.^5–7^

Although the molecular mechanism of insulin resistance is not fully understood,^8^ inositol phosphoglycan, one of the intracellular mediators of the insulin signal, has been shown to correlate with insulin sensitivity in type 2 diabetes mellitus.^9,10^ The increased urinary excretion of inositol phosphoglycan affects blood glucose levels^11^ and also occurs in patients with polycystic ovary syndrome and insulin resistance,^12^ who have been successfully treated with myo-inositol and folic acid.^13^ The action of insulin in patients with polycystic ovary syndrome is also improved by the administration of d-chiro-inositol.^14^ Therefore, it is speculated that the excretion of inositol phosphoglycan contributes to the insulin resistance associated with polycystic ovary syndrome. These studies are relevant to the possible therapeutic use of myo-inositol supplementation for the prevention of GDM. One study has suggested that insulin resistance in gestational diabetes could be improved by the administration of inositol, as in polycystic ovary syndrome,^14^ and several studies have shown that myo-inositol supplementation increases the action of endogenous insulin.^10,11,15,16^ These studies imply that myo-inositol supplementation can be used therapeutically to prevent GDM.

However, the sample sizes in these studies have been relatively small, so it is necessary to collate all the available evidence that myo-inositol supplementation can prevent GDM. The aim of this meta-analysis was to determine whether myo-inositol supplementation increases the action of endogenous insulin, which is usually measured by markers of insulin resistance, such as the homeostasis model assessment (HOMA) of insulin resistance.

## METHODS

### Study Selection

PubMed (January 1966–February 2015), the Cochrane Library (2015 Issue 2), EMBASE (January 1974–February 2015), and Web of Science (2015 Issue 2) were searched for relevant studies. The search terms used were “gestational,” “diabetes mellitus,” and “myo-inositol,” and both medical subject headings and free texts were screened. Relevant publications were identified, and their “related articles” and their citations were also scanned. Additional searches were performed, mainly by reviewing the relevant review articles. The publications were not limited to specific languages.

### Inclusion and Exclusion Criteria

The articles were critically reviewed by 2 reviewers for their eligibility for our meta-analysis. Only controlled studies of myo-inositol supplementation in pregnant women were selected. Outcome measures were required, which usually included the incidence of gestational diabetes, offspring birthweight, fasting glucose oral glucose tolerance test (OGTT), 1 h OGTT, 2 h OGTT, and related complications.

### Data Extraction and Quality Evaluation

The data were independently extracted and cross-checked by 2 researchers. The methodological quality of the studies was assessed using the methods recommended by the Cochrane collaboration. These mainly involved the randomization process, allocation concealment, blinding, follow-up, baseline characters, and analytical method.

### Statistical Analysis

The available data on the outcome measures for all the trials were extracted, pooled, and analyzed. The *χ*^2^ statistic was used to evaluate the heterogeneity of the trials and the *I*^2^ statistic to assess the extent of inconsistency. Odds ratio (OR), risk ratio (RR), and mean difference (MD), and their respective 95% confidence intervals (95% CIs) were estimated with a fixed-effects or random-effects meta-analysis model. All statistical analyses were performed with Review Manager (RevMan version 5.3). A subgroup analysis was used to clarify the different diagnoses of the related complications.

The meta-analysis was reported according to the PRISMA statement. All the included studies declared that the study was approved by local ethics committee, and our meta-analysis itself did not involve any ethics issues.

## RESULTS

### Literature Search

**FIGURE 1:**
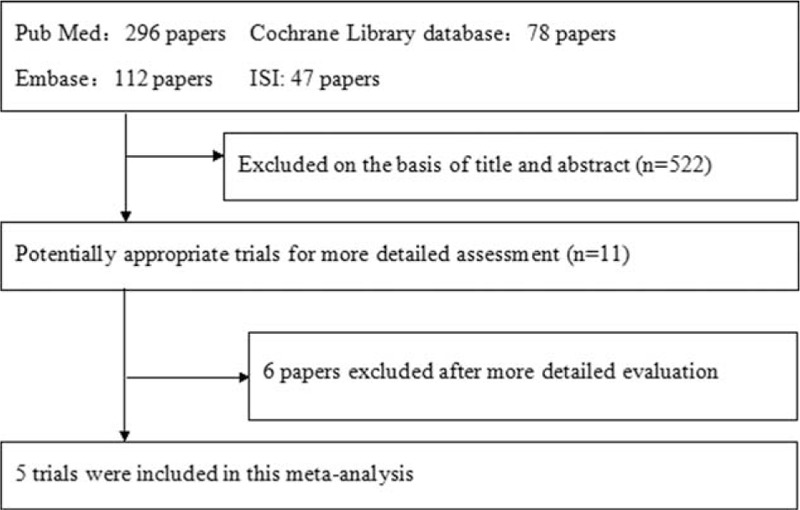
Flow chart of trial selection from initial literature search to final studies inclusion.

The preliminary search identified 533 potentially relevant articles. Some articles were excluded after further evaluation because they were irrelevant to the proposed interventions, were reviews, lacked control studies, or were duplicates. In total, 11 articles were assessed further, and 6 of them were excluded when the full text was read. Ultimately, 5 trials in which the participants underwent hysterectomy were retrieved from the electronic databases.^10–14^Figure 1 shows the flow chart for study selection, from the initial results of the publication search to the final inclusion or exclusion of the articles.

### Characteristics and Methodological Quality of the Included Studies

Table 1 gives specific information on the articles evaluated, including the study type, participants, interventions, and outcome measures. In total, 513 participants were included in these 5 studies. Among the studies, 2 studies administrated myo-inositol 2 mg per day,^10,13^ and 3 studies administrated myo-inositol 4 mg per day.^11,12,14^ And 3 studies adopted myo-inositol throughout the pregnancy,^12–14^ while 1 study adopted myo-inositol at 12 to 13 weeks of pregnancy,^10^ and the other one adopted it when GDM was diagnosed.^11^Table 2 summarizes the methodological quality of the included studies, which was assessed with the methods recommended by the Cochrane Handbook 5.0.2.

**TABLE 1:**
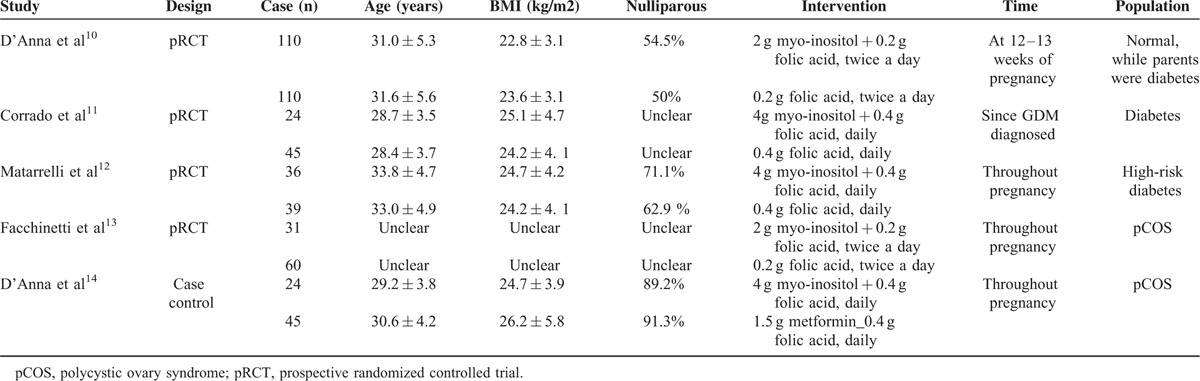
Baseline characteristic of patients in the included trials

**TABLE 2:**
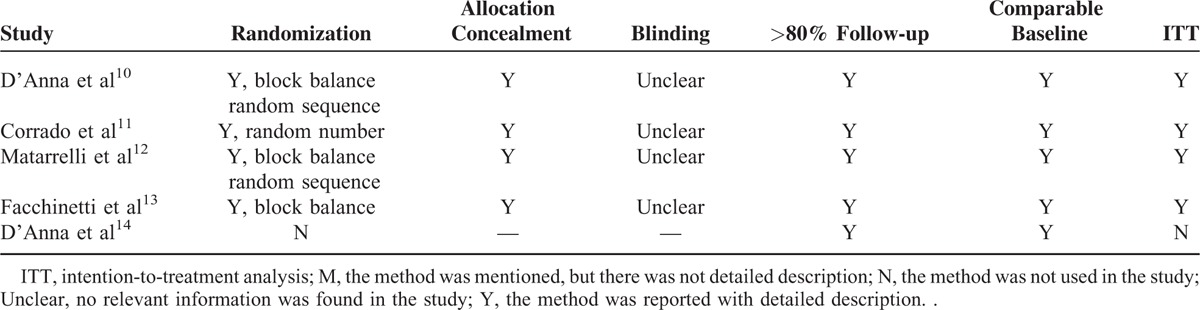
Quality Assessment of Included Trials

### Incidence of GDM

**FIGURE 2:**
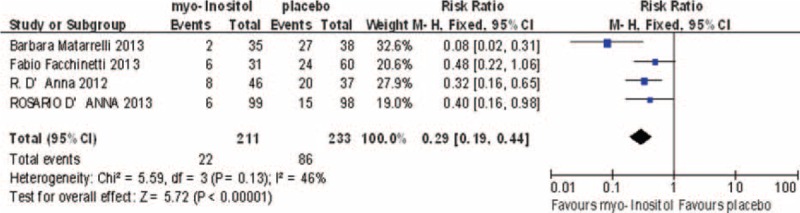
Meta-analysis result of the incidence of gestational diabetes between the groups.

Four studies involving 444 participants reported the incidence of GDM. The studies were not highly heterogeneous (*I*^2^ = 46%). In the random-effects model, there was a statistically significant difference between the myo-inositol treated group and the control group (RR 0.29; 95% CI, 0.19–0.44; *P* < 0.00001; Fig. 2).

### Birth Weight

**FIGURE 3:**
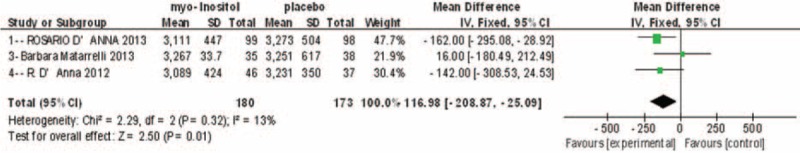
Meta-analysis result of birth weight between the groups.

Three studies involving 353 participants reported birth weight, and were homogeneous (*I*^2^ = 13%). In the fixed-effects model, there was a statistically significant difference between the myo-inositol treated and control groups (MD, –116.98; 95% CI, –208.87 to –25.09; *P* = 0.01; Fig. 3).

### Fasting OGTT

**FIGURE 4:**
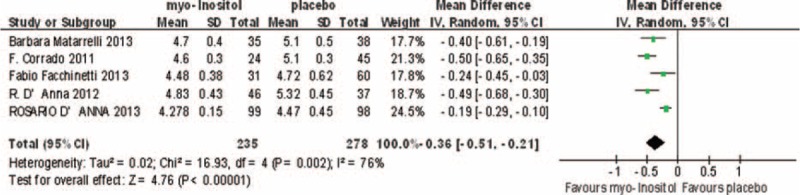
Meta-analysis result of fasting glucose OGTT between the groups.

Four studies involving 422 participants reported fasting OGTT results, and they were highly heterogeneous (*I*^2^ = 76%). In the random-effects model, there was a statistically significant difference between the myo-inositol treated and control groups (MD, –0.36; 95% CI, –0.51 to –0.21; *P* < 0.0001; Fig. 4).

### One-hour OGTT

**FIGURE 5:**
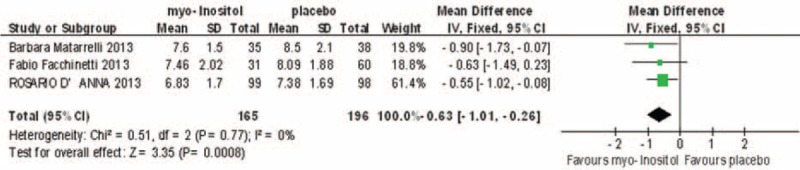
Meta-analysis result of 1-h glucose OGTT between the groups.

Three studies involving 361 participants reported 1-h OGTT results, and were homogenous (*I*^2^ = 0%). In the fixed-effects model, there was a statistically significant difference between the myo-inositol treated and control groups (MD, –0.63; 95% CI, –1.01 to –0.26; *P* = 0.002; Fig. 5).

### Two-hour OGTT

**FIGURE 6:**
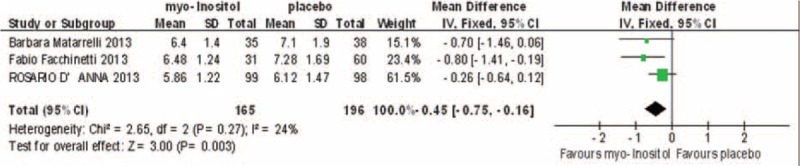
Meta-analysis result of 2-h glucose OGTT between the groups.

Three studies involving 361 participants reported 2-h OGTT results, which showed some heterogeneity (*I*^2^ = 24%). In the fixed-effects model, there was a statistically significant difference between the myo-inositol treated and control groups (MD, –0.45; 95% CI, –0.75 to –0.16; *P* = 0.002; Fig. 6).

### Incidence of GDM-Related Complications

Three studies reported GDM-related complications, including respiratory distress syndrome, shoulder dystocia, neonatal hypoglycemia, macrosomia, polyhydramnios, and preterm delivery. A pooled analysis in the fixed-effects model (*I*^2^ = 36%) showed a significant difference in the overall incidence of complications (OR 0.28; 95% CI, 0.14–0.58; *P* < 0.001).

**FIGURE 7:**
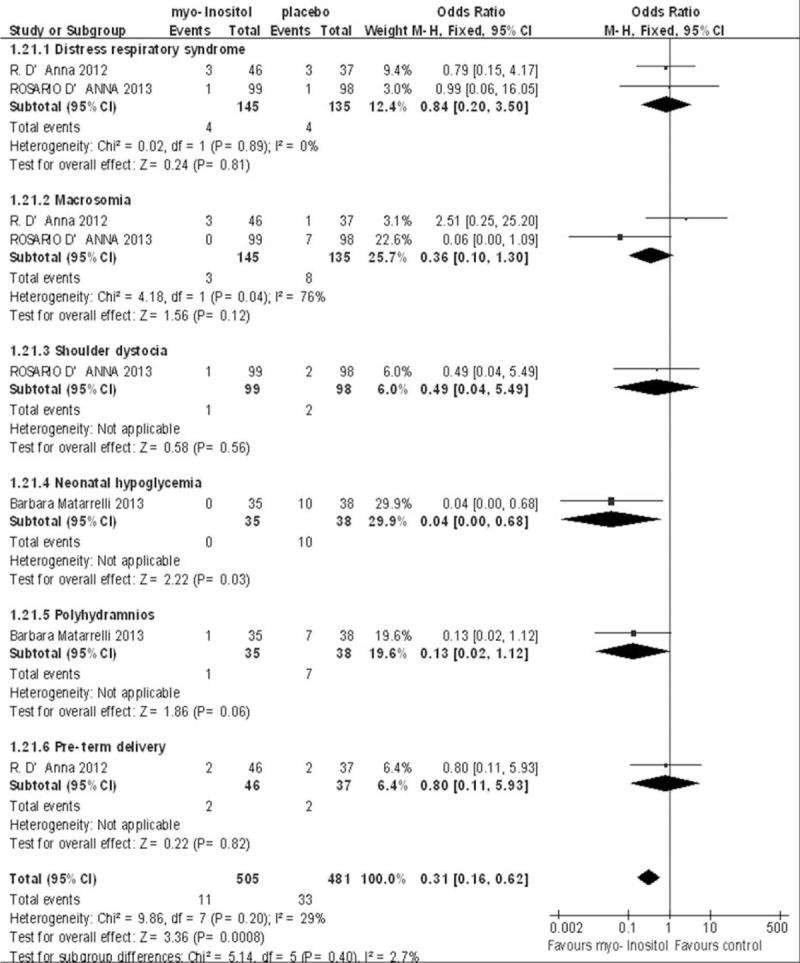
Meta-analysis result of the incidence of gestational diabetes mellitus related complications.

Two studies reported data on respiratory distress syndrome, and the results showed no significant difference between the myo-inositol supplementation and e control groups (OR 0.84; 95% CI, 0.20–3.50; *P* = 0.89). Two studies included data on macrosomia and showed no significant difference between the 2 groups (OR 0.36; 95% CI, 0.10–0.30; *P* = 0.05). Only 1 study reported shoulder dystocia, neonatal hypoglycemia, polyhydramnios, and preterm delivery, and showed that myo-inositol supplementation reduced the incidence of neonatal hypoglycemia. Although it also tended to reduce the incidence of polyhydramnios, the difference was not statistically significant. There was no difference between the 2 groups in terms of the incidence of shoulder dystocia or preterm delivery (Fig. 7).

## DISCUSSION

The global prevalence of hyperglycemia in pregnancy is 16.9%, and more than 90% of these women are estimated to reside in low- and middle-income countries.^17^

Many factors and changes would exist in or lead to GDM. Because pancreatic beta cells lack antioxidant-scavenging enzymes, they are very vulnerable to reactive oxygen. So, the endoplasmic reticulum (ER) stress response is likely activated in them, to some extent leading to mitochondrial dysfunction, and fuel-stimulated insulin release would be reduced. Further, a study of the molecular mechanisms underlying insulin secretion showed that the leakage of intracellular Ca^2+^ via the mutant type 2 ryanodine receptor (RyR2), a Ca^2+^ release channel on the ER of pancreatic beta cells, also plays a crucial role in ER stress response and insulin secretion reduction.^18^ Mechanism relevant to adrenergic system supports the proposition that the downregulation of the β2-adrenergic receptor is associated with age-related impaired glucose tolerance.^19^ In the last 2 decades, circumstantial evidence has suggested that gestational diabetes originates, at least partly, in the intrauterine and neonatal environments. During pregnancy, the mother's metabolism is extensively altered to support fetal development and growth. Insulin resistance becomes particularly severe during the second half of pregnancy, when insulin secretion increases by 200% to 250% to maintain euglycemia.^20^ If insufficient insulin is secreted, hyperglycemia and GDM develop. The adverse programming of beta cells may also be transmitted to subsequent generations.

The prevention of GDM is extremely important because high-glucose concentrations are associated with teratogenesis, which can affect the fetal conformation, function, and development, and has long-term adverse effects on the offspring. The children born of women with GDM are also at an increased risk of macrosomia and birth defects,^21^ and they are more likely to develop childhood obesity, glucose intolerance in early adulthood,^22,23^ and gestational diabetes themselves.^24^ Therefore, glucose control is important in pregnancy. The major findings of this meta-analysis are that compared with the control group, women supplemented with myo-inositol showed a reduced incidence of GDM.

Fasting OGTT, 1-h OGTT, and 2-h OGTT were all reduced in the myo-inositol group. Interestingly, the results of 1 study also suggested that the expression of adipocytokines was downregulated in the control group and upregulated in the myo-inositol treated group, and that adipocytokine levels were significantly higher in the myo-inositol treated group than in the control group. It has been reported that adipocytokine levels correlate negatively with glucose and insulin concentrations,^25,26^ and are reduced in the insulin-resistant state. Therefore, adipocytokine levels could be used as a proxy to assess basal insulin levels and insulin sensitivity.^27^ These data are consistent with the results of the HOMA model assessment. One study reported that the HOMA-measured insulin resistance were reduced by 50% in the myo-inositol treated patients,^11^ and a previous study in patients with polycystic ovary syndrome reported similar results.^28^ The researchers believed that this reduction in insulin resistance was predominantly attributable to the adipocytokine adiponectin, because it was the only adipocyte-derived hormone downregulated in insulin-resistant patients.^11^

Myo-inositol may have an important role as a mediator of the insulin signaling cascade,^29^ and the coupling of insulin to specific receptors stimulates the intracellular transport of inositol phosphoglycan.^30^ Therefore, it can be inferred that myo-inositol directly activates acetyl-CoA-carboxylase stimulating lipogenesis or plays a role as a precursor of d-chiro-inositol containing inositol phosphoglycan. However, no strong experimental evidence supports this inference. Inositol is reported to improve insulin sensitivity because it acts as a second messenger, which may exert an insulin-like effect on metabolic enzymes.^27^ The biochemical mechanism underlying the regulation of glucose metabolism by myo-inositol supplementation requires further study.

This meta-analysis also showed that the mean birth weight was significantly lower in the myo-inositol treated group. Our pooled analysis also showed that myo-inositol supplementation reduced the incidence of GDM-related complications, including respiratory distress syndrome, shoulder dystocia, neonatal hypoglycemia, macrosomia, polyhydramnios, and preterm delivery. However, in the subanalysis, only the difference in the incidence of neonatal hypoglycemia was statistically significant. Myo-inositol supplementation tended to reduce the incidence of macrosomia and polyhydramnios, but not statistically significantly. It has been shown that supplementary inositol benefits preterm infants with respiratory distress syndrome, reducing adverse neonatal outcomes.^29^ A multicenter study with a larger study population is required to explore whether myo-inositol supplementation reduces the incidence of adverse outcomes of GDM.

There are some weaknesses in the present evidence. In all the studies included, myo-inositol supplementation was open label, and the failure to blind either the women recipients or the drug provider in any of the studies had a negative effect. One of the trials included was a retrospective case–control study and may have increased the likelihood of random assignments. This meta-analysis was also limited by differences in the inclusion criteria used in the studies and by variations in the components of the interventions, including metformin and folic acid. The women in the intervention groups also used more than 1 intervention, such as dietary control and folic acid, which would have confounded the beneficial effects of myo-inositol. The studies were limited in their reporting of the proportions of women who complied with the intervention, which could have had a major influence on the observed effect size. Another limitation is the generalizability of the meta-analysis. All the subjects were Caucasian women from Italy, and no other ethnic group was represented, so it remains unclear whether the findings are applicable to pregnant women in other countries. In a study of polycystic ovary syndrome, Nestler et al^25^ first reported the beneficial effects of myo-inositol in Venezuelan women, but these findings were not confirmed in a later study^22^ of Caucasian women in the USA. The main weakness of our meta-analysis was that only 3 studies evaluated the adverse obstetric outcomes of GDM, such as perinatal death, macrosomia, shoulder dystocia, bone fracture, nerve palsy, elective cesarean, early delivery, and emergency cesarean section. Therefore, a multicenter study with a larger study population is required to evaluate the risk of adverse effects in the high-risk group.

The beneficial effects of myo-inositol supplementation on GDM appear promising. The optimal dose, frequency, and type of inositol isomer are still unclear, and the effects of different forms and various doses on GDM must be identified. It is likely that myo-inositol supplementation will be cost-effective, and it is an attractive option because it is readily available throughout the world. Therefore, the effects of myo-inositol supplementation must be evaluated in large, multicenter, randomized controlled trials, involving individuals from different ethnic backgrounds.

## CONCLUSIONS

On the basis of current evidence, myo-inositol supplementation reduces the development of GDM, although this conclusion requires further evaluation in large-scale, multicenter, blinded, randomized controlled trials.
